# Identifying dysfunctional crosstalk of pathways in various regions of Alzheimer's disease brains

**DOI:** 10.1186/1752-0509-4-S2-S11

**Published:** 2010-09-13

**Authors:** Zhi-Ping Liu, Yong Wang, Xiang-Sun Zhang, Luonan Chen

**Affiliations:** 1 Key Laboratory of Systems Biology and SIBS-Novo Nordisk Translational Research Centre for PreDiabetes, Shanghai Institutes for Biological Sciences, Chinese Academy of Sciences, Shanghai 200031, China; 2 Academy of Mathematics and Systems Science, Chinese Academy of Sciences, Beijing 100190, China; 3 Institute of Industrial Science, The University of Tokyo, Tokyo 153-8505, Japan

## Abstract

**Background:**

Alzheimer's disease (AD) is a major neurodegenerative disorder leading to amnesia, cognitive impairment and dementia in the elderly. Usually this type of lesions results from dysfunctional protein cooperations in the biological pathways. In addition, AD progression is known to occur in different brain regions with particular features. Thus identification and analysis of crosstalk among dysregulated pathways as well as identification of their clusters in various diseased brain regions are expected to provide deep insights into the pathogenetic mechanism.

**Results:**

Here we propose a network-based systems biology approach to detect the crosstalks among AD related pathways, as well as their dysfunctions in the six brain regions of AD patients. Through constructing a network of pathways, the relationships among AD pathway and its neighbor pathways are systematically investigated and visually presented by their intersections. We found that the significance degree of pathways related to the fatal disorders and the pathway overlapping strength can indicate the impacts of these neighbored pathways to AD development. Furthermore, the crosstalks among pathways reveal some evidence that the neighbor pathways of AD pathway closely cooperate and play important tasks in the AD progression.

**Conclusions:**

Our study identifies the common and distinct features of the dysfunctional crosstalk of pathways in various AD brain regions. The global pathway crosstalk network and the clusters of relevant pathways of AD provide evidence of cooperativity among pathways for potential pathogenesis of the neuron complex disease.

## Background

Recently, systems biology approaches such as network-based methods have been successfully applied to elucidate the mechanism of diseases [1-3]. For instance, human transcriptome and interactome can be integrated to bridge the gap between genotype and phenotype [4-6]. Alzheimer's disease (AD) is a debilitating neurodegenerative progression and fatal disease. The genetic mechanisms of AD are far from being clear although there are several popular hypotheses about its pathogenesis [7-9]. The availability and integration of high-throughput gene expression data [10] and the genome-wide protein-protein interaction may shed new lights on AD study.

Biological processes in a cell are carried out through interactions among many proteins [11], which are functional units and generally achieve specific tasks cooperatively [2]. In other words, the activities of proteins are organized into modules that form many pathways. Often, genes in the same pathway are activated together and thus exhibit similar gene expression patterns. And genes with similar expression profiles are more likely to encode interacting proteins to coordinately achieve a particular function [11,12]. Regarding to AD, a key challenge is to identify the biological processes or signaling pathways which play significant roles in the development of disease. Here, we aim to integrate the interrelated transcriptomic and interactomic information together to investigate AD related pathways.

The system complexity is coming from not only the cooperations of proteins in the form of pathways but also the interactions of pathways, i.e., the crosstalks of these pathways [13,14]. Here crosstalk is defined as the fact that two pathways are likely to interact with or influence each other. Crosstalk between pathways provides more complex nonlinear responses to combinations of dysfunctions [15,16]. To study pathogenesis of AD, some studies show that the gradual cognition decline may correspond to disease progression in different brain regions responsible for certain independent functions individually [17]. Neuronal loss occurred in AD often begins from a deep brain region, i.e. entorhinal cortex, and then spread to the hippocampus [10,17], a key part of brain controlling memories and movements. From the systematic perspective, analysis of AD related bio-molecular interaction networks in different brain regions will improve the understanding of the complexity of molecular pathways underlying AD phenotypes and will help to uncover the dynamic processes of disease progression.

In this paper, we focus on the spatial pathway clusters in different AD brain regions and the crosstalks among pathways by integrating protein-protein interaction (PPI) and gene expression data. Specifically we define dysfunctions of pathway as the responsive scores of these involved protein interactions from control cases to disease cases. Currently, several computational methods [2] have been proposed to detect active pathways, and those methods are designed to find differentially expressed gene sets [18,19] and the corresponding protein sets, and then these differentially scored proteins with all their reported interactions are taken as dysregulated pathways [20,21]. However, we note that the information on protein interaction gives not only an outline of protein relationship [22,23], but also the possibility of dysfunctional cooperations in disease. The known interactions of these disease proteins can then be used to detect the specific dysfunctions by combining with other information, e.g., the expression of genes and the correlation between them [24-26].

To this end, we propose a network-based analysis for the crosstalks among AD related pathways in different AD brain regions by integrating protein-protein interactions and region-specific gene expression profiles. Initially, we build a protein network of pathways by collecting KEGG AD pathway and its neighbor pathways. Then, we propose a new scoring scheme to define the dysfunctions of protein interactions by combining gene expression and co-expression information. The crosstalks of these dysfunctional pathways are identified by their overlapping relationship in six AD brain regions. In addition, we cluster AD related pathways into groups by their significance of overlapping and the related pathways are sorted by their scores of dysregulation. Finally, we demonstrate that the brains process comprehensive crosstalk between pathways on the protein level due to AD progression in various regions.

## Results

### Clusters of AD related pathways

**Figure 1:**
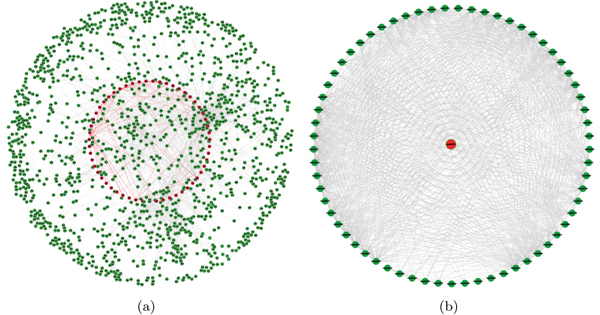
**Network of pathways.** Subfigure (a) shows the ensemble protein-protein interaction network in which the proteins of KEGG AD pathway and its neighbor pathways are involved. AD pathway is highlighted in red. Subfigure (b) shows the topological linkages among the AD pathway and its neighbor pathways, where node denotes pathway and there is an edge if two pathways contain at least one common protein.

We built a network of pathways by integrating the pathway information from KEGG [27] and the protein-protein interaction network. The gene set of KEGG AD pathway and that of all its neighbor pathways were mapped to the ensemble protein-protein interaction network (see Methods). Figure 1(a)  shows the protein-protein interaction network where the genes in the KEGG AD pathway are highlighted in red. Then we plotted the pathway as nodes and linked two pathways by an edge if the two pathways have overlapping genes. Figure 1(b) shows the global topology linkages of AD pathway and its neighbor pathways. The red node in Figure 1(b) corresponds with the red part of AD pathway in Figure 1(a).

**Figure 2:**
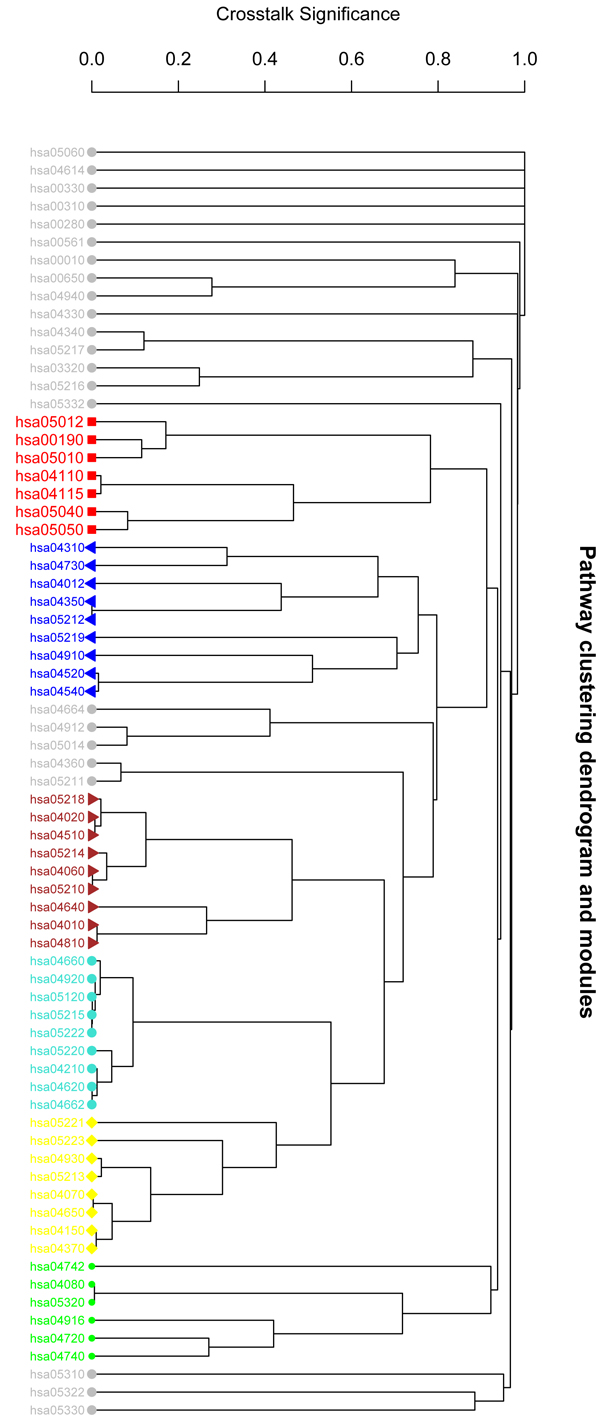
**Hierarchical clustering of pathways in EC region.** Different modules are represented by different colors. The red one represents the cluster including KEGG AD pathway (hsa:05010). The grey ones are those pathways which can not be clearly clustered into groups. The detailed clustering results are listed in Table 1.

After weighting the network edges by integrating gene expression and co-expression data in six AD brain regions (see Methods), we can evaluate if the interactions among pathways are significant. The overlapping score and its significance can be used as a measurement of close relationship among them. Then a similarity matrix of these pathways was built and used to group the pathways into clusters. Figure 2 gives the clustering result in these pathways based on their interaction significance in EC region. We then did the similar clustering for these pathways in the other five regions. Table 1 lists the results of the cluster in which KEGG AD pathway is grouped. In Figure 2, different colored clusters describe different modules. The red cluster is the module of KEGG AD pathway (ID: hsa05010) and its closest interaction pathways. The modules in grey color are the part of pathways which cannot be grouped to any individual cluster distinctly. We found that several pathways of neurodegenerative diseases were grouped together, such as Parkinson's disease (hsa05012), Huntington's disease (hsa05040) and Dentatorubropallidoluysian atrophy (hsa05050). The significant interaction between two pathways provides evidence for their close interrelations. This result indicates the close relationship between these neuron diseases. Pathway modules in other brain regions are similar to those in EC region (results are shown in Table 1). These results not only provide us evidence for the close relationship between AD and related neuron diseases [28], but also validate the effectiveness of our method. Moreover, we also identified some closely related pathways with AD in the same cluster, e.g., Oxidative phosphorylation (hsa:00190), p53 signaling pathway (hsa04115), and Apoptosis (hsa04210). Genetic evidences support that oxidative phosphorylation is closely related to AD [29]. Functionally important reductions in oxidative phosphorylation enzyme activities appear to occur in AD and may be related to *β*-amyloid accumulation, which is the main phenotype in AD patient brains [29]. Thus this kind of dysfunction defects could play an important role in the pathophysiology of AD. The critical role of p53 is proved by the fact that it is mutated in a very large fraction of tumors [30]. It is an important transcriptional activator whose activity is regulated by phosphorylation. p53 is also known as a potential biomarker for AD for its special alternation presented in A*β* accumulation [30]. The signaling pathway can cause the cell to enter apoptosis process (pathway ID: hsa04210). The brains of AD patients contain dying neurons displaying apoptosis. The close relationship between pathways of apoptosis and AD indicates that apoptosis plays an important role in the progression of the disease. From this viewpoint, the therapies of AD need consider the apoptosis features of the neuron cell. The clusters of AD related pathways provide us a global view of the relationships among these pathways. The category also clearly indicates the close dysfunctional pathways related to AD in various brain regions.

**Table 1:** Clusters of KEGG AD pathway (hsa05010) in different brain regions

Region	Pathway ID	Size	Edge	Node	P-value	Description
EC	hsa00190	137	7	12	0.115269	Oxidative phosphorylation
	hsa04110	119	344	101	1.000000	Cell cycle
	hsa04115	69	72	46	0.146277	p53 signaling pathway
	hsa05012	136	25	26	0.130562	Parkinson's disease
	hsa05040	32	27	21	0.207717	Huntington's disease
	hsa05050	15	10	11	0.213708	Dentatorubropallidoluysian atrophy (DRPLA)

HIP	hsa04080	302	106	128	0.815693	Neuroactive ligand-receptor interaction
	hsa04115	69	72	46	0.362224	p53 signaling pathway
	hsa04720	72	37	32	0.117635	Long-term potentiation
	hsa04730	75	41	38	0.025842	Long-term depression
	hsa05050	15	10	11	0.347488	Dentatorubropallidoluysian atrophy (DRPLA)

MTG	hsa04360	129	100	72	0.094750	Axon guidance
	hsa04370	74	54	41	0.672299	VEGF signaling pathway
	hsa05014	56	35	28	0.853204	Amyotrophic lateral sclerosis (ALS)
	hsa05040	32	27	21	0.707504	Huntington's disease
	hsa05050	15	10	11	0.719474	Dentatorubropallidoluysian atrophy (DRPLA)

PC	hsa04210	89	156	72	0.195914	Apoptosis
	hsa04730	75	41	38	0.880031	Long-term depression
	hsa05040	32	27	21	0.084885	Huntington's disease
	hsa05050	15	10	11	0.056018	Dentatorubropallidoluysian atrophy (DRPLA)

SFG	hsa04010	274	403	188	0.082078	MAPK signaling pathway
	hsa04360	129	100	72	0.094750	Axon guidance
	hsa04370	74	54	41	0.344348	VEGF signaling pathway
	hsa05014	56	35	28	0.257644	Amyotrophic lateral sclerosis (ALS)
	hsa05040	32	27	21	0.505385	Huntington's disease

VCX	hsa00190	137	7	12	0.197734	Oxidative phosphorylation
	hsa05012	136	25	26	0.291280	Parkinson's disease
	hsa05040	32	27	21	0.518223	Huntington's disease
	hsa05050	15	10	11	0.516887	Dentatorubropallidoluysian atrophy (DRPLA)

“Size” is the number of genes contained in KEGG gene sets. “Edge” and “Node” represent the number of edges and nodes of these pathways in our protein-protein interaction network with gene expression information. “P-value” gives the p-value of dysregulation score of every pathway in the cluster. “Description” gives the pathway name.

### Dysfunctional crosstalk between pathways

From the clusters, we identified the pathway modules with close relationships. The crosstalk of dysregulated pathways exist not only in the same clusters of pathway, but also exist within all its neighbor pathways. As to these AD related pathways, we defined the dysfunction score and its significance criterion (see Methods). We individually identified the significance of their dysfunctions in six AD brain regions. Table 2 lists the top five ranked pathways in every region. The dysfunction score of every pathway neighbored to AD pathway represents the activation status during the AD progression in the form of pathway of protein interactions. We found that Apoptosis (hsa04210), Notch signaling pathway (hsa04330), Wnt signaling pathway (hsa04310), and Cytokine-cytokine receptor interactionare (hsa04060) are the most significant pathways in the six regions. From the rank, we gave a quantitative measure of the dysfunctional activation of these neighbor pathways when AD pathway performs its dysfunctions of neuron-toxic processes. Among the pathways in the clusters containing AD pathway, we can find that some of them are also the most significant pathways. Apoptosis is not only with high interaction significance with AD pathway, but also with high dysfunction significance itself. The result gives more evidence for the strong relationship between apoptosis and AD process [31]. Especially, Notching signaling pathway is twice ranked as the first in the six regions. The pathway is known to be crucial in communication between the cells, which involves important processes during embryonic and adult life [32]. When developing medicines for AD, the side effect for Notch signaling pathway is an important factor because it may inhibit the drug targets. Wnt signaling pathway often involves in Ca^2^+ signaling, which leads to transient increases in cytoplasmic free calcium that subsequently activates the calcium kinase and the phosphatase calcineurin [27]. It is now known that the Ca^2^+ is often crucial to AD, which is regarded as the calcium hypothesis [33]. If the calcium level is not properly controlled, it would lead to neuron cell dysfunction and death. Calcium flows into brain cells in an unregulated way and affects cell function and survival. Cytokines are also crucial to cell death. Proinflammatory cytokines may lead to neuron death and dysfunction by variant mechanisms. Inhibition of cytokines has been tested as a therapy method for the treatment of AD [34]. In summary, the significant neighbor pathways indicate the dysfunctional crosstalk between those pathways and AD pathway. The critical influences to AD were identified from the neighbor pathways.

**Table 2:** Rank of AD related pathways

Region	Pathway ID	Size	Edge	Node	P-value	Description
EC	hsa04210	89	156	72	0.000001	Apoptosis
	hsa04060	279	259	220	0.000002	Cytokine-cytokine receptor interaction
	hsa04620	102	110	69	0.000695	Toll-like receptor signaling pathway
	hsa05220	75	181	69	0.003802	Chronic myeloid leukemia
	hsa05120	68	54	40	0.003899	Epithelial cell signaling in Helicobacter pylori infection

HIP	hsa04310	152	152	83	0.000000	Wnt signaling pathway
	hsa05214	65	113	52	0.000000	Glioma
	hsa04060	279	259	220	0.000001	Cytokine-cytokine receptor interaction
	hsa04012	87	200	71	0.000002	ErbB signaling pathway
	hsa05215	90	181	72	0.000002	Prostate cancer

MTG	hsa04330	46	59	34	0.000000	Notch signaling pathway
	hsa05120	68	54	40	0.000000	Epithelial cell signaling in Helicobacter pylori infection
	hsa04310	152	152	83	0.000003	Wnt signaling pathway
	hsa04520	78	105	59	0.000024	Adherens junction
	hsa04210	89	156	72	0.000388	Apoptosis

PC	hsa04060	279	259	220	0.000000	Cytokine-cytokine receptor interaction
	hsa04640	87	49	62	0.000000	Hematopoietic cell lineage
	hsa04115	69	72	46	0.000049	p53 signaling pathway
	hsa04210	89	156	72	0.000689	Apoptosis
	hsa05217	55	33	26	0.001264	Basal cell carcinoma

SFG	hsa04330	46	59	34	0.000000	Notch signaling pathway
	hsa04510	203	340	150	0.000000	Focal adhesion
	hsa04920	67	79	39	0.000002	Adipocytokine signaling pathway
	hsa04210	89	156	72	0.000009	Apoptosis
	hsa05120	68	54	40	0.000190	Epithelial cell signaling in Helicobacter pylori infection

VCX	hsa04060	279	259	220	0.000000	Cytokine-cytokine receptor interaction
	hsa04640	87	49	62	0.000000	Hematopoietic cell lineage
	hsa04340	57	14	11	0.000121	Hedgehog signaling pathway
	hsa04510	203	340	150	0.000279	Focal adhesion
	hsa04662	65	93	51	0.002533	B cell receptor signaling pathway

The most significant 5 pathways in six AD brain regions are ranked individually by “P-value”, which gives the p-value of dysregulation of the pathways. “Size” presents the number of genes contained in KEGG gene sets. “Edge” and “Node” represent the number of edges and nodes of these pathways.

**Figure 3:**
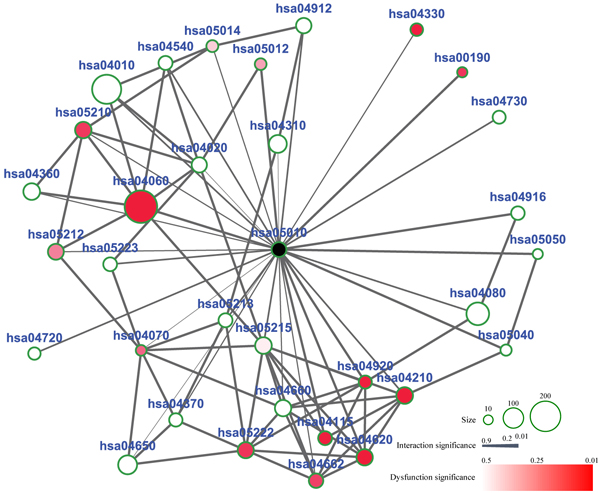
**Dysfunctional crosstalk of AD related pathways.** The center dark node is the KEGG AD pathway and other nodes are its neighbor pathways. The size of the nodes represents the number of proteins contained in the pathways. The width of the edges represents the significance of dysfunctional relationship between the two pathways in EC region. The color of the nodes represents the dysfunction significance of these neighbor pathways in EC region.

**Figure 4:**
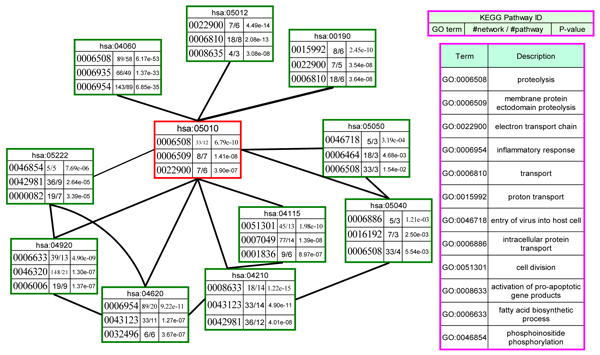
**The top three GO functions which are enriched in the crosstalks among the dysfunctional pathways.** Significantly enriched GO biological processes are identified in every pathway respectively. The nodes are the pathways with significantly enriched GO annotations. Pathway ID, GO terms, number of the GO terms in the network/pathway, and hypergeometric p-values are presented in nodes. The description of the node and GO terms are shown on the right part. The one in the center is the AD pathway.

The rank of dysfunctional significance implies the degree of inflections from neighbor pathways to AD pathway. In EC region, there are several other dysfunctionally significant neighbors in addition to the top 5 pathways in Table 2. The crosstalk status between AD related pathways are shown in Figure 3, where the center node is the AD pathway and the others are its neighbor pathways. The interaction significance of two pathways is represented by the width of their edge. The dysfunction significance of a pathway is shown by the corresponding gradient node color. The node size represents the number of proteins involved in the pathway. From Figure 3, we can clearly detect the two indices of describing the crosstalk between pathways, i.e., the intra-pathway dysfunction score and inter-pathway interaction strength. Oxidative phosphorylation pathway (hsa00190) significantly interacts with AD pathway (hsa05010), Cytokine-cytokine receptor interaction pathway (hsa04060), Calcium signaling pathway (hsa04020), and p53 signaling pathway (hsa04115). The calcium signaling pathway provides evidence for the dysfunctional relationship between calcium with AD mechanism [33]. Interestingly, some of them also have significant interactions with each other which constitute a module formation in these neighbor pathways. We note that the pathways in the module are mainly contained in the same cluster of Figure 2. The pathway clusters in neighbor pathways are also the cooperative group with dysfunctional crosstalk to AD pathway. This implies the complexity of the neurodegenearative disease and provides new hypotheses of AD. As to the crosstalk of GO [35] functional relationships between these pathways, we identified the accumulative hypergeometric significant GO biological processes in every pathway. The functional enrichment among proteins in one pathway is defined as:

where *n* is the number of nodes in the network, *ƒ* is the number of proteins annotated with a particular GO function, *m* is the number of proteins involved in the pathway and *k* is the frequency of the GO term. We identified the GO function enrichment of the pathways in six brain regions respectively. The results of the top three GO terms in part of the pathways are shown in Figure 4. The most significant biological processes in AD pathway are proteolysis (GO:0006508), membrane protein ectodomain proteolysis (GO:0006509) and electron transport chain (GO:0022900). The functions correspond to the main processes of A*ß* accumulation in AD progression. The significant functions of neighbor pathways provide a flow of transporting of molecules (macromolecules, small molecules, protons and other ions) of substances into, out of, within, or between cells (GO:0006810, GO:0015992). Other pathways introduce one or more phosphate groups into a phosphoinositide (GO:0046854). Especially, Apoptosis pathway (hsa04210) enriches the conversion of proteins, and induces or sustains apoptosis to an active form (GO:0008633). From the significant GO enrichments, we know the crosstalk of GO biological processes during the disease development between the pathways in various brain regions. The most popular hypotheses of AD mechanism is the amyloid cascade hypothesis and tau hypothesis, and the disease is caused by the accumulation of abnormally folded A*ß* and tau proteins in the brain [29]. The GO significance among these pathways provides high correlated functions in these pathways which provide implications that the closely related pathways are crucial to the dysfunction of AD pathway during the disease progression.

## Discussion

In this work, we identified the dysfunctional crosstalk of AD related pathways in different brain regions based on a novel network-based method. In the AD protein network, we first built the network of pathways by integrating KEGG pathways information and protein-protein ensemble interactions. The crosstalk of pathways underlying their interaction significance and their dysfunctional score were detected by corresponding gene transciptome information in six disease brain regions respectively. The analysis of dysfunctional crosstalk with AD pathway is based on the pathway clusters and all its neighbor pathways as well as their clusters related to AD pathway. Some of the identified dysfunctional crosstalk between the pathways are consistent with our knowledge for AD. Some of them provides valuable alternatives for AD mechanism, especially from the pathway relationship perspective. The interaction of these dysfunctional pathways provides more insights for the AD progression in various brain regions.

### Closely related pathways of AD

When identifying the crosstalk between the pathways in AD disease brain regions, we only chose AD pathway and its neighbor pathways. Temporarily, many important pathways in KEGG were not considered in this study if they do not contain overlapped genes with the AD pathway. In other words, we only studied the neighbor pathways with direct interaction with AD pathway. The strategy is based on the straightforward assumption that the nearest neighbor pathways imply the close relationship with AD pathway. We acknowledge that the pathways with indirect interactions with AD pathway might also transfer potential dysfunctions to AD and then are important for the pathway clusters and dysfunctional processes. These pathways should be further explored in future.

Additionally, our method can be similarly applied to all the annotated pathways. Moreover, clustering of those AD related pathways into pathway clusters provide more information. The similar clusters in different brain regions show that the crosstalks among these pathways are relatively consistent in different brain tissues. This indicates the common or identical dysfunctions in various AD brain regions. The clusters containing AD pathway imply that the neurodegenerative diseases have close relationships, which provide evidence for the effectiveness of our method.

### Scoring schemes

We collected AD related gene sets of pathway and linked the genes by our integrated PPI network. Then we mapped the region-specific gene expression profiling to the protein interaction network. The edges are scored by the Fisher's method by combining the statistical significance p-values of differential expression and co-expression. The dysfunctions between two proteins are simultaneously evaluated by their gene expression and correlation in various disease brain regions individually. The edge-scoring scheme considers the differential expression from the control to the disease and the correlation information in the disease cases. The meta-analysis is applied to integrate p-values between the interacting proteins with independence assumption [36]. In the light of performing functions by protein interactions, dysfunctions of protein interaction are represented from the expression and correlation level of two proteins. Then the dysfunctional importance of the pathways are their dysregulation underlying the interactions and the quantitative significance. We defined the dysregulation score of pathways by summarizing the scores of contained interactions. And the overlapping score between two pathways is the summary of edge scores on their common interactions. Moreover, the scores have been normalized by transforming into statistical significance values [37]. The dysfunction scores of the pathways and the interaction scores between the pathways are used for identifying the crosstalk.

### Crosstalk among the pathways

We clustered AD and its neighbor pathways by their statistical significance of overlapping scores. The interaction significance are represented as a distance matrix. From hierarchical clustering, we identified the pathway correlations and detected their relationship in six AD brain regions respectively. We identified the crosstalk of these pathways in the clustered groups as well as with their neighbor pathways, especially with AD pathway. The crosstalk between pathways other than AD pathway might also contribute to AD by their cooperative dysfunctions. In this paper, we considered the nearest neighbor pathways with AD pathway. A future research direction is to identify the dysfunction of indirect interaction between one pathway and AD pathway. Moreover, the crosstalk with AD pathway is not only related to the interaction, but also related to their own dysfunctions. We identified the significant dysfunctions of the pathways in every brain regions. Some of them are those with high interaction significance with AD pathway. These pathways are important for the dysfunctional crosstalk with AD process during the development in various brain regions. We identified the crosstalk among these pathways and provided detailed analysis of the interactive dysfunctions with AD pathway. We also identified the biological processes enrichments underlying these interacted pathways. The GO functional linkages of these pathways provided more implications for their dysfunctional crosstalk.

### Regional feature of crosstalk

We identified the pathway clusters in six functionally important brain regions respectively. There are some regional features of the crosstalk, such as the difference of the rank of pathways in Table 2. We focused on both the similarity among the pathway clusters and the specificity in every cluster. The mechanism of AD dysfunction might underlie the common features, while the regional feature would be the biomarker for the specific diseased tissues. The comparison of pathway clusters and then the dysfunctional difference in different regions gives more knowledge of the status of protein cooperativity in specific brain region. The decline of normal activity abilities is known to be controlled by different brain regions [17], and then the specificity would also provide detailed information for the dysfunctions.

## Conclusion

In this paper, a network-based approach was used to analyze the crosstalks among AD related pathways. The clusters of pathways were identified and the related pathways were ranked by their dysfunctional scores. The dysfunctional crosstalks of pathways are found and analyzed in six AD brain regions. The results are consistent with our prior knowledge of AD. The crosstalk of pathways presents new alternative insights for AD pathology. Our work shows that comprehensive and system-wide analysis provides evidence for neurodegenerative AD disorder and complements the traditional component-based approaches.

## Methods

### Data sources

**Figure 5:**
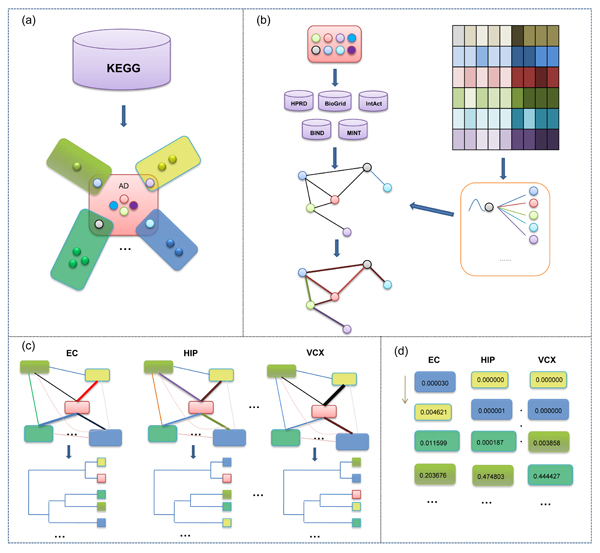
**Schematic representation of identifying and analyzing the crosstalk of dysfunctional pathways in six AD brain regions.** (a) Gene sets of AD pathway and its neighbor pathways are identified from KEGG; (b) Networking the proteins by the integrated PPI from five known databases and mapping the region specific gene expression profiling information to the network of pathways; (c) Identifying the relationships among AD related pathways in six brain region and clustering them into groups respectively; (d) Ranking these pathways by dysfunctional score.

We downloaded the AD pathway and all its neighbor pathways from KEGG. Here the neighbor pathways are defined as those pathways which have at least one overlapping gene with AD pathway. In total there are 77 neighbor pathways in KEGG intersecting with AD pathway (see Additional file 1). Figure 5(a) shows the schematic representation for this procedure. We then mapped all the genes to proteins from NCBI [38] and represented them by their NCBI Entrez Gene IDs. Then we applied a voting method to construct an ensemble protein-protein interaction network by integrating five existing PPI databases in human, i.e., HPRD [39], BIND [40], BioGrid [41], IntAct [42], and MINT [43]. Roughly we selected those interactions contained in at least three of the five databases. The comprehensive protein-protein interaction network contains 7,533 nodes and 22,345 edges.

We extracted the gene expression profile data on AD patients with normal controls from [10], which were deposited in NCBI GEO [44] database (ID:GSE5281). The expression data were used to study AD progression in six functionally and anatomically distinct brains regions [10], including entorhinal cortex (EC), hippocampus (HIP), middle temporal gyrus (MTG), posterior cingulate cortex (PC), superior frontal gyrus (SFG), and visual cortex (VCX). We preprocessed the CEL source files by RMA algorithm with defaulted parameters in R bioconductor package [45]. Probe sets were mapped to NCBI entrez genes using DAVID [46]. If there are multiple probe sets that correspond to the same gene, the expression values of those probe sets are averaged. The expression dataset contains 22,283 probes and, as a result, leads to 13,932 genes. Temporarily, these genes without corresponding proteins in the integrated PPI network and corresponding gene in the expression experiments were not included in the analysis as well as the isolated proteins in every pathways. This ended up with an KEGG AD pathway with 54 nodes and 79 edges and 70 neighbor pathways. The network of all these pathways contains 1,401 proteins and 2,888 interactions (see Additional file 2). All the source data are in the version of September 2008.

### Mapping gene expression to PPI network

To measure the differential expression status of gene, we used Welch's t-test to calculate the p-value from the expression data of control and disease cases. To determine the co-expressed significance of a gene pair in disease cases, we used the Pearson correlated coefficient test to calculate the p-value. Then we mapped those p-values to the nodes and edges in the protein-protein interaction network (Figure 5(b)). Fisher's method [36] is used to define a function as the combination of statistical significance of an interaction by a scoring scheme in the following formula:

where diff(*x*) and diff(*y*) are differential expression assessments of gene *x* and gene *y*, respectively. corr(*x*‚*y*) represents their correlation between gene *x* and gene *y*. *f* is a general data integration method that can handle multiple data sources differing in statistical power. Where *k* = 3, *p*_1_ and *p*_2_ are the p-values of differential expression of two nodes, *p*_3_ is the p-value of their co-expression. The gene profiles for different specific regions of AD were mapped respectively and we obtained six condition-based weighted protein interaction networks (see Figure 5(b)).

### Interaction significance between pathways

We define an interaction function to evaluate the significance of all non-empty overlaps between two pathways in different brain regions individually, i.e.,

where *P_i_* and *P_j_* are two pathways, and *O_ĳ_* is their overlapping. Specifically, the interaction score between two pathways is estimated by their overlapping status of weighted pathways in the following formula:

The overlapping score is the summation of the scores of overlapping edges between pathways. To estimate the significance of the overlapping between different disease brain regions, we random sample 10^6^ times of the same size two pathways in the edges of pathway network and calculate their overlapping scores. The frequency larger than *C* is regarded as the interaction significance p-value. From the scores, a distance matrix of these pathways is built and used to clustering these pathways into pathway groups. Hierarchical clustering is implemented for grouping the pathways into clusters by using the dynamicTreeCut package in R [45] (see Figure 5(c)).

### Dysregulation of related pathways

To define the dysfunction of a pathway *P* in various regions of AD, we summarize all the scores of edges *S*(*e*) of every pathway, i.e.,

. To estimate a p-value for significance of this pathway, we iteratively compute similar scores 10^6^ times on randomly generated pathways of the same size as that of pathway *P*. The frequency of scores that are larger than *Sp* is used as the significance p-value of pathway *P* to describe its dysregulation in one specific brain region. In every region, we get a ranked list of dysfunctional pathways (see Figure 5(d)). The detailed analysis of crosstalk of dysfunctional relationship among pathways is then investigated, especially that with AD pathway.

## Abbreviations

AD: Alzheimer's disease; EC: entorhinal cortex; HIP: hippocampus; MTG: middle temporal gyrus; PC: posterior cingulated cortex; SFG: superior frontal gyrus; VCX: visual cortex; PPI: protein-protein interaction; GO: gene ontology; NCBI: national center for biotechnology information; KEGG: Kyoto encyclopedia of genes and genomes; HPRD: human protein reference database; MINT: molecular interactions database; BIND: biomolecular interaction network database; BioGrid: biological general repository for interaction datasets; IntAct: molecular interaction database; GEO: gene expression omnibus; RMA: Robust multi-array average; DAVID: Database for annotation, visualization and integrated discovery

## Competing interests

The authors declare that they have no competing interests.

## Authors' contributions

ZPL and LC conceived the research. ZPL designed and performed the study and YW improved it. XSZ gave valuable suggestions and improvements. LC and XSZ supervised the project. All authors wrote and approved the manuscript.

## Supplementary Material

Additional file 1 Description of AD related pathways. Click here for file

Additional file 2 Source data of the constructed protein network of pathways. Click here for file
